# Supplementary figures and images for: Simulating flow induced migration in vascular remodelling
Source: PLoS Comput Biol. 2020 Aug 21;16(8):e1007874. doi: 10.1371/journal.pcbi.1007874 (PMC7478591; doi:10.1371/journal.pcbi.1007874)

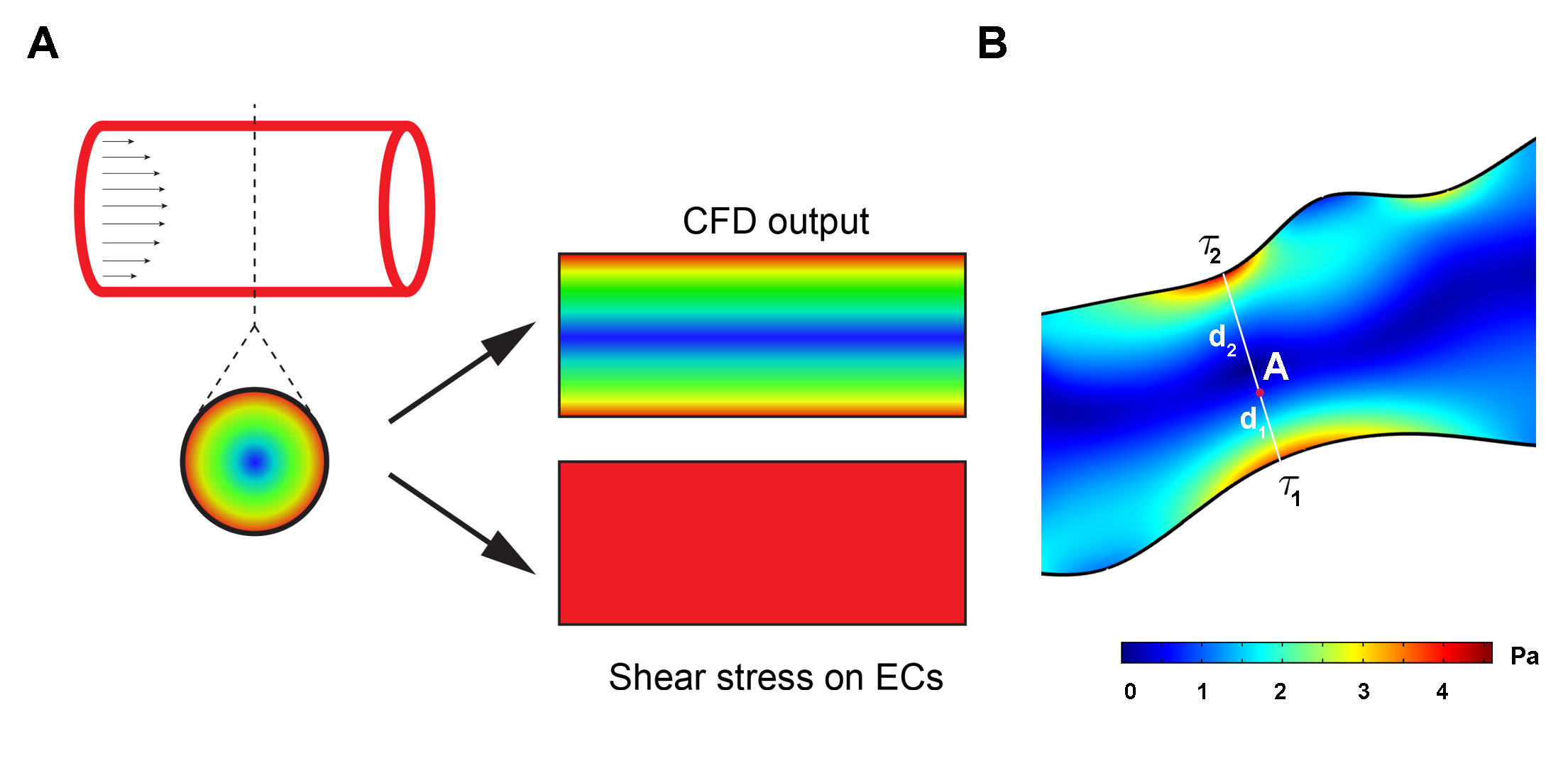

Supplement: S1 Fig — A) Shear stress levels within the fluid and along the vessel wall. B) Calculating the shear stress level for each point throughout the vasculature from interpolation of the nearest walls’ shear stress levels. (TIF) [file pcbi.1007874.s001.tif]

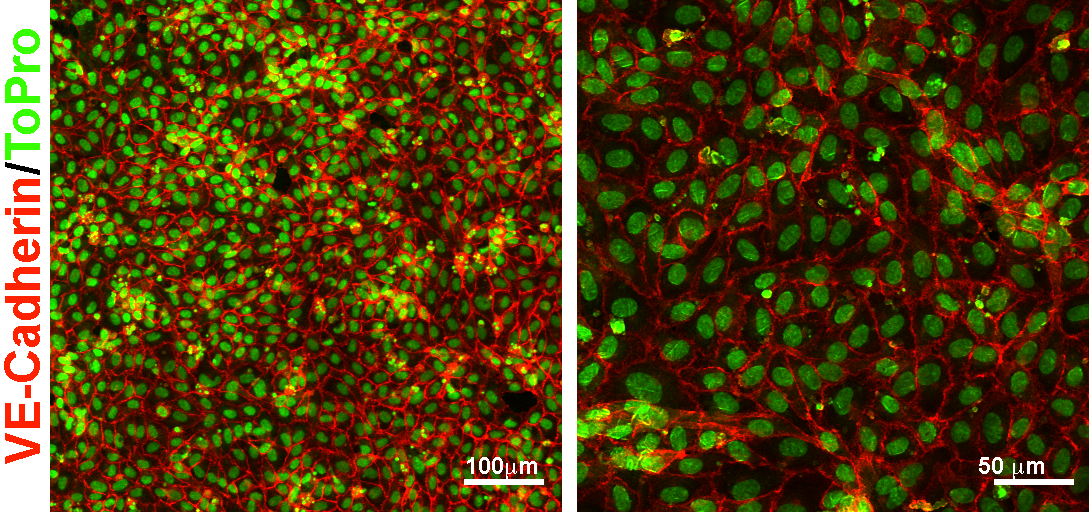

Supplement: S2 Fig — Cells were seeded, as for shear stress experiments, in our chambers and then fix and stained for VE-Cadherin. Monolayers showed continuous and straight cell-cell junctions within the endothelial layer. Nuclei were labelled with ToPro. (TIF) [file pcbi.1007874.s002.tif]

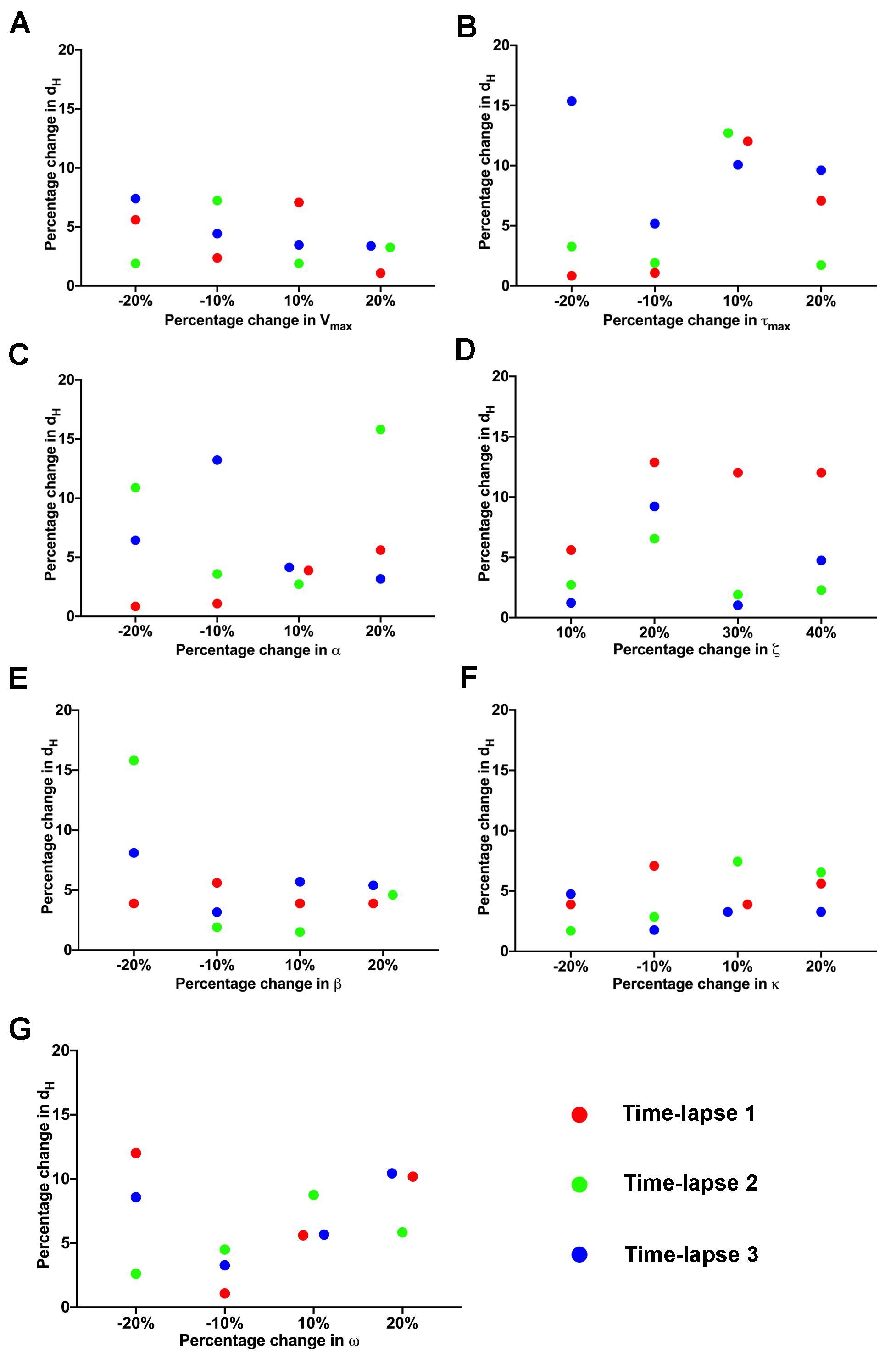

Supplement: S3 Fig — Parameters were varied to identify how errors in each parameter would affect the final prediction for A) Vmax which is the maximum velocity of migration at τmax, B) τmax which is the value of shear stress at which the maximum migration occurs, C) α which is the importance of velocity vectors in alignment of adjacent agents, D) ζ which is the importance of shear stress gradients, E) β which is the importance of body forces between vascular agents, F) κ which is the importance of body forces from avascular agents on vascular agents, and G) ω which is the remodelling force. (TIF) [file pcbi.1007874.s003.tif]
