# Supplementary material for: Simulating flow induced migration in vascular remodelling
Source: PLoS Comput Biol. 2020 Aug 21;16(8):e1007874. doi: 10.1371/journal.pcbi.1007874 (PMC7478591; doi:10.1371/journal.pcbi.1007874)
Supplement: Attachment — Submitted filename: Response to Reviewers.docx [file pcbi.1007874.s009.docx]

Dear Dr Scott Diamond,

We were pleased to read that our manuscript “Simulating flow induced migration in vascular remodelling” (PCOMPBIOL-D-20-00575) was of considerable interest to the editor and reviewers. We highly appreciated the input from the reviewers and feel that this has significantly improved the manuscript. Please find a detailed point by point response to the reviewer’s concerns as follows.

Two new supplemental figures (S2 Figure and S3 Figure) were added to the manuscript to address requested new data. S2 Figure addresses the maturity of ECs before the shear and S3 Figure represents the sensitivity analysis performed on the model parameters. Furthermore, the text has been changed in some sections to address points that were unclear to the reviewers. These are highlighted in red.

We sincerely hope that the added experimental data and information provided will answer to the requests of the reviewers and will be considered appropriate by you and the reviewers. We look forward to receiving the feedback on our revision.

Kindest regards,

Elizabeth Jones

--------------------------------------------------------------------------- 
Response to reviewers

Reviewer #1: In this manuscript, Tabibian et al. design, develop and validate a model of vascular remodeling during embryogenesis. The model is based on experimental data, collected both in vivo and in vitro to define values and ranges of flow-dependent hemodynamic conditions, cell shape and migration. The model predictions are challenged against real experimental observation and the relevance of different parameters is evaluated.
The results obtained are overall interesting, with a well-crafted set of experiments to describe the evolution of both vascular and non-vascular components, with reference to the overall development of the body.

We thank the reviewer for his/her comments and enthusiasm.

The manuscript should be accepted after the noted changes:

1. The distinction between vascular and non-vascular agents is clear from the perspective of the model, much less for the biology. Vascular agents are endothelial cells, but then what are non-vascular agents? The authors should clarify if their categorisation is purely based on the imaging, or it has a biological meaning, which then can be used in the interpretation of the results.

The non-vascular agents are mesenchymal cells. In the yolk sac, these avascular regions have spares mesenchymal cells, separated by significant amounts of ECM. This is also partially why we choose to expand the interaction radii of the avascular agents, rather than having them proliferate. Most of this space, in effect, becomes ECM devoid of cells. This was shown by TEM by Hirakow & Hiruma in 1981. We have expanded on this in the text and referenced this article.

On p. 4-5 (line 134-136), we added the sentence: “The avascular spaces of the embryo consistent of mesenchymal cells, surrounded by significant amounts of extracellular matrix (ECM).”

Later in the same paragraph (line 143-144), we added: “This, in part, represents growth due to additional deposition of ECM rather than purely due to cell proliferation.”

2. HUVEC monolayers used in flow experiments should be better characterised. I am missing some IF staining confirming that the reconstituted tissue is fully mature at the time of flow onset. VE-cadherin distribution and density evaluation should be enough.

As suggested, we have stained the cells and added this data into supplemental materials (S2 Figure). We agree that this strengthens the manuscript since we label only a subset of all cells for the tracking experiments.

3. It looks like some figures and quantifications do not report the number of independent experiments.

These were represented as individual data points in the figures. We have now added explicit statement regarding the number of repetitions to the legend.

4. In my experience the cell tracker staining can affect the migration of HUVEC. Limiting the % of cells receiving the staining surely helps, but due to the relevance of these experimental data, I suggest to have a control evaluation. A reference wound healing experiment could be used to test whether in these experimental conditions collective cell migration is preserved.

This is an excellent suggestion. We chose not to perform a wound healing experiment, however, because we specifically want cells to migrate as a sheet. In our opinion, the velocity when neighbour-neighbour interaction are missing will be significantly higher than when cell-cell contact are present, possibly just due to steric inhibition. Thus, migration into a cell-free area would have different kinetics that migrating within an area full of cells.

To investigate the effect of our cell tracker, we have instead repeated the migration experiments for 0.1 Pa with a second, completely unrelated, cell label. In this case, we used a membrane dye rather than a cytoplasmic dye. We used Vybrant DiI (Invitrogen, V22885) for this purpose. Our results (shown in the figure bellow) show a collective migration with lower velocity in this case (6 μm/h in contrast to 10 μm/h for cell tracker).

Although these results do show that the cell label affects migration rate, this will not affect our model because we only keep the shape of the shear stress vs migration velocity curve (i.e. the bell shape), but alter the location of Vmax, as well as the value of Vmax. For this reason, we have chosen not to include the above results in the manuscript. We can, however, include them as a supplemental if the reviewer believes that this is important.

Reviewer #2: This paper presents a computational model of vascular remodeling based on in-vitro and in-vivo experimental data in the embryo yolk sac. Specifically, a simple agent-based model takes into account shear stress, cell migration and EC density during embryonic development. It is well-known that shear stress is a key regulator of vascular remodeling, but the details of the mechanisms are not known. Overall, this is an interesting study with potential relevance to vascular development. However, there are a number of issues with the approach that should be addressed.

1) This model is based on a previous agent-based model and has been slightly improved by including a term for shear stress to include its effect on ECs migration. However, it is not clear from the results how this term improves the prediction of migration by ECs.

This is addressed in Figure 7. In our model, we use a system similar to Grégorie et al. but we use the neighbor-neighbor interaction to define only the direction of migration and not both the direction and magnitude as Grégorie et al. had done. In our model, the magnitude is defined by the shear stress levels.

We have made this clearer in the text by adding the following sentences on p. 9 (line 279-282): “Our model overall uses cell-cell interactions to define the angle of migration and the shear stress levels to define the magnitude of the velocity of migration. The cell-cell interactions for the angle were previously developed by Grégorie et al. [26].”

And then later in the paragraph (line 285-288): “a model where agents migrate in random directions but using shear stress-induced velocity magnitudes (Fig 7B), a model a model where direction of flow and cell-cell interactions define the angle of vascular agents' migration, as previously described by Grégoire et al. (Fig 7C)."

2) This model has many theoretical parameters that cannot be determined experimentally. If it is not possible to experimentally determine these parameters at least a sensitivity analysis should be performed to show the sensitivity of the results to these parameters.

As suggested by the reviewer, we have included sensitivity analysis for our variables. The results were added as S3 Figure and described on p.9 (line 268-277).

3) Some experimental results (Fig. 6 at hr 4) are not in agreement with computational results. The authors should further discuss the discrepancies.

The same experimental results (Fig 6, at 4hrs) appear as panel A of Figure 8. We do discuss the results at that point.

4) The authors should cite the seminal works of Secomb and Pries, who pioneered the area of shear-induced vascular remodeling.

Indeed. We have added the sentence to introduction (p2 line 25-26) stating: “Shear stress-driven pruning, contraction, and enlargement of vessel within a network was first introduced by Pries and Secomb.”

Later in the discussion, on p 11. (line 334-338): “Models by Secomb and Pries include influences from non-vascular regions in terms of changes in oxygen partial pressure, but they did not model the physical forces created by these cells as they expand.”

5) Collective migration is a result of cell crowding (which the authors reproduce as repulsive forces), but also cell-cell adhesion (which is not considered in the model). Would the results be improved if there were some tendency for cells to “drag” each other due to adhesive forces?

These are indeed included within the cell-cell adhesive forces, as developed by Grégoire et al. In this model, if the cells are too close, they repel but if they are too far apart, they attract, similar to a Lennard-Jones potential.

We have made this clearer in the text by adding the following sentences to p. 6 (line 177-179): “Agents that are too close are repelled from each other, however agents that are too far are attracted, thereby creating both repulsive and adhesive forces between agents that are required for cells to migrate as a sheet.”

6) Most importantly, it is not clear that the presented 2D model reproduces accurately the 3D tissue being considered. In reality, cells in the avascular space have an extra degree of freedom to move into or out of the 2D domain. Similarly, cells in the vessel really don’t have a fixed boundary preventing migration laterally. They are bounded by basement membrane around the circumference, but can move with the same freedom around the circumference or along the axis of the vessel. To accommodate the reduction to 2 dimensions, the authors made some critical assumptions about the interactions between the endothelial cells and the surrounding tissue at the boundaries, which are very suspect. Thus, it is unlikely that the presented model truly reproduces the mechanisms present in vivo.

The growth and migration of the avascular region is indeed 3 dimensional. Based on the movies produced by Cui et al., most of the migration of endothelial cells is in the direction of flow and therefore the 2D model is, in our opinion, appropriate. We agree, however, that it can never completely model the in vivo, especially in regions with more complicated flow patterns. But, definitely, there are limitations to our model. Most importantly, better modelling of the border between vascular and avascular regions are needed. Ours is the first model to include physical forces between growing tissues and remodeling vessels and I hope that our will work will highlight the need for more refined solid-fluid boundary models in tissues. This is definitely an area for future work within our group, and I hope for others in the field.
